# Adenomatoid odontogenic tumor: Case series of 14 with wide range of clinical presentation

**DOI:** 10.4317/jced.54216

**Published:** 2017-11-01

**Authors:** Fatima Al-Shimari, Srinivasa Chandra, Dolphine Oda

**Affiliations:** 1 Department of Oral & Maxillofacial Surgery, University of Washington School of Dentistry, Seattle, Washington

## Abstract

**Background:**

To present 14 cases of adenomatoid odontogenic tumor (AOT), highlighting their clinical, radiographic, and histologic characteristics.

**Material and Methods:**

Fourteen cases of AOT were retrieved from the archives of the Oral Pathology Biopsy Service (OPBS). Clinical, radiologic, and histologic findings are described.

**Results:**

Fourteen AOT cases were reviewed, of which 12 were intraosseous and two were peripheral (gingiva). The cases came from eight females and six males with an age range of 11–30. Of the 12 intraosseous cases, nine were follicular (associated with impacted teeth), while three were extra-follicular (present between teeth). Six of the 12 cases were in the maxilla, and the other six were in the mandible. The two peripheral cases presented as nodules on the buccal gingiva of the anterior maxillary teeth. Radiographically, all 12 follicular and extra-follicular cases were unilocular radiolucencies; of those, only one had specks of radiopacity. Histologically, all specimens were similar in morphology, demonstrating a varied degree of duct-like structures, epithelial spheres, spindle-shaped epithelial cells, calcifications, and a thick capsule. The two peripheral cases had no capsule.

**Conclusions:**

AOT usually affects patients under 20 years of age, with a female to male ratio close to 2:1. Presentation in the anterior maxilla is almost twice as common as in the anterior mandible. Radiographically, AOT presents as a unilocular radiolucency more commonly associated with impacted teeth, simulating a dentigerous cyst. We present 14 new cases of AOT (nine follicular, three extra-follicular, and two peripheral) with discussions of their clinical, radiographic, and histological features.

** Key words:**Adenomatoid, odontogenic, tumor.

## Introduction

Adenomatoid odontogenic tumor (AOT) is an uncommon benign neoplasm of odontogenic epithelial origin accounting for less than 3% of all odontogenic tumors (1-3). Benign and slow-growing, it is believed to originate from the remnants of the dental lamina or the enamel organ (1,4-6). In 1969, Philipsen *et al.* proposed the name “adenomatoid odontogenic tumor,” which was later adopted by the World Health Organization with the understanding that this lesion has a benign and non-aggressive behavior (2-5,7,8). AOT has three clinical presentations: intra-osseous (associated with impacted teeth and also known as “follicular”; 70% of cases); intra-osseous (present between erupted teeth and also known as “extra-follicular”; 25% of cases; and on the gingiva (extra-osseous, also known as “peripheral”; 5% of cases) (1). The anterior maxilla and the mandible are the most common locations for AOT, with maxilla being almost twice as likely as the mandible (3,9). The follicular variety is more commonly associated with impacted maxillary canines, which account for 60% of cases (1,3,10). It is important to note, however, that AOT cases have also been described in the posterior mandible and maxilla, but rarely beyond the premolars. About 53% of AOT cases occur in the anterior maxilla, while 9% occur in the maxillary premolar region (11). About 2% of AOT cases occur in the molar region (11). AOT is more common in females, with a female to male ratio of close to 2:1 (1,3,12). Almost 70% of AOT cases occur in the second decade of life, with an age range of 10-19, and rarely occur in patients above the age of 30.1 Clinically, the size of the tumor is usually small around 1-3 cm in diameter; occasionally, however, sizes larger than 3 cm are described (1,11). In general, AOT cases are usually mildly expansile. Occasionally, they are asymptomatic (2,4,12). Radiographically, AOT cases usually present as well-defined to corticated unilocular radiolucent lesions; (9,11,13) about 10% demonstrate some degree of calcifications (14). Histologically, AOT is made up of epithelial cells arranged in strands of spindle-shaped cells, epithelial spheres/whorls, and cuboidal epithelial cells arranged in duct-like structures with or without calcifications (6,11,13). The nature of the calcifications ranges from nonspecific to cementum-like globules. Sometimes, globules of a homogenous eosinophilic material are present that may represent amyloid (6,15). These tumors are supported by a thick, fibrous connective tissue capsule which makes separation of the lesion from the tooth and the surrounding bone easy, allowing the clinician to occasionally save the impacted tooth in follicular AOT cases. The treatment of choice is conservative surgical removal through simple curettage or enucleation (4,9). Recurrence has been described, but it is exceedingly rare (3,4,9,16).

## Material and Methods

Fourteen cases were retrieved from the archives of the Oral Pathology Biopsy Service (OPBS), Department of Oral Surgery, at a major research university. The radiographic and clinical findings were reviewed by an oral surgeon, and the hematoxylin- and eosin-stained (H&E-stained) glass slides were reviewed by an oral pathologist. Statistical testing to determine any significant difference in our comparisons was not possible due to the small size of our series. IRB approval was obtained to perform this study.

## Results

-Clinical Findings: General

 Table 1 summarizes the main clinical and radiographic features of the 14 AOT cases retrieved from the OPBS between the years 2005 and 2013, during which 45,667 biopsies were processed. The 14 cases accounted for 0.03% of the cases read at the OPBS in this time period. Twelve (86%) of the 14 AOT cases were in bone (64% follicular and 22% extra-follicular), and two (14%) occurred on the gingiva only (peripheral). Of the 14 patients, six were males (43%) and eight were females (57%) with an age range of 11-30 years (mean = 16). Eight of the cases were in the maxilla (57%) and six were in the mandible (43%); if the peripheral cases are excluded, 6 cases were in the maxilla and 6 in the mandible. Five of the 14 cases (36%) involved impacted canines. Twelve of the 14 patients (86%) were under 20 years of age; the other two were 28 and 30 years of age. Eleven (79%) of the 14 cases were in the anterior maxilla and mandible and three were in the premolar area. The three cases in the premolar area were in the mandible (cases 1, 7 and 13). Eleven of the 12 intraosseous cases presented with swelling; case #4 was asymptomatic with no evidence of swelling. It was discovered as an incidental finding, simulating a lateral periodontal cyst.

**Table 1:**
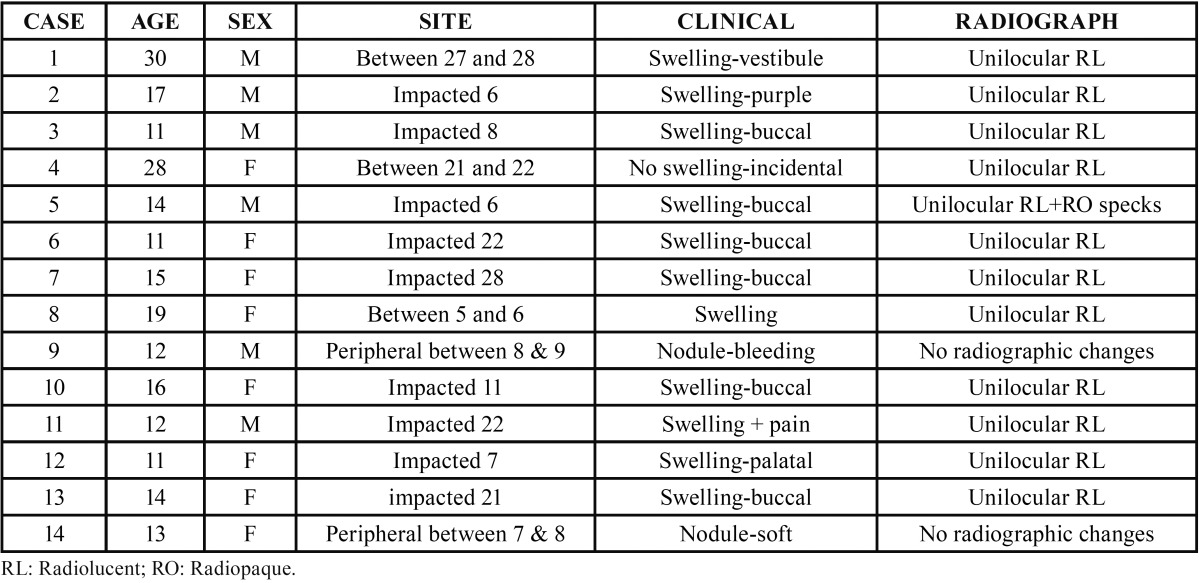
Summary of Clinical, Radiographic, and Histologic Features of 14 AOT cases.

**Figure 1:**
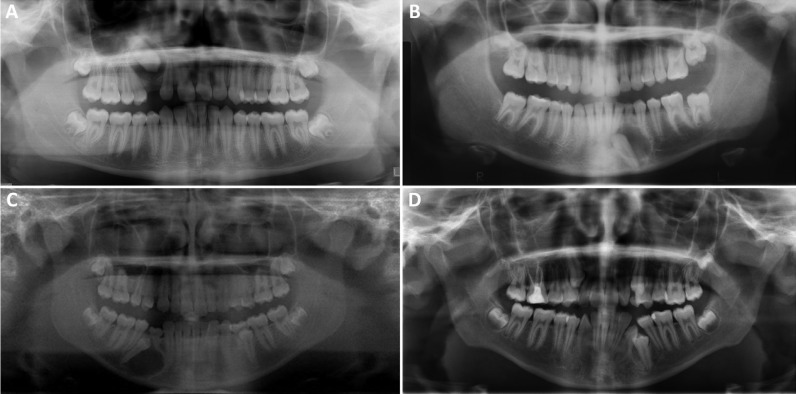
Radiological features of some of the nine Follicular AOT cases. A: This panoramic image demonstrates a well-demarcated unilocular radiolucency with specks of radiopacity associated with impacted left maxillary canine. B: This panoramic image demonstrates a well-demarcated unilocular radiolucency associated with impacted left mandibular canine. C: This panoramic radiograph demonstrates a well-demarcated unilocular radiolucency associated with impacted right mandibular first premolar pushing teeth apart. D: This panoramic radiograph demonstrates a well-demarcated unilocular radiolucency associated with impacted left mandibular second premolar. Note how it interrupts the eruption of the adjacent left mandibular canine.

-Clinical Findings: Follicular AOT (associated with impacted teeth; Figs. 1A-1D)

Nine of the AOT cases were follicular in type: cases 2-3, 5-7, and 10-13. The age range for the follicular AOT cases was 11-17 years (mean = 13) with female to male ratio of 1.3:1. Figures 1A-1D are radiographic examples of four of the follicular AOT cases: cases 5, 6, 7, and 13. Of the nine follicular AOT cases, five (56%) were associated with impacted canines: cases 2, 5, 6, 10, and 11. Cases 5 and 6 are depicted in Figures 1A and 1B, respectively. Three of the five impacted canines were in the maxilla (cases 2, 5, and 10) and two were in the mandible (cases 6 and 11). The other four follicular AOT cases were associated with impacted teeth #s 7, 8, 21, and 28. Two examples are depicted in Figures 1C (case 7) and 1D (case 13). Five of the nine follicular AOT cases were in the maxilla (56%) and four were in the mandible (44%). All nine cases presented with some degree of clinical swelling; eight reported buccal swelling and case 12 reported palatal swelling. Other clinical changes included pain associated with case 11 and a change in color associated with case 2, which was described as clinically purple.

**Figure 2:**
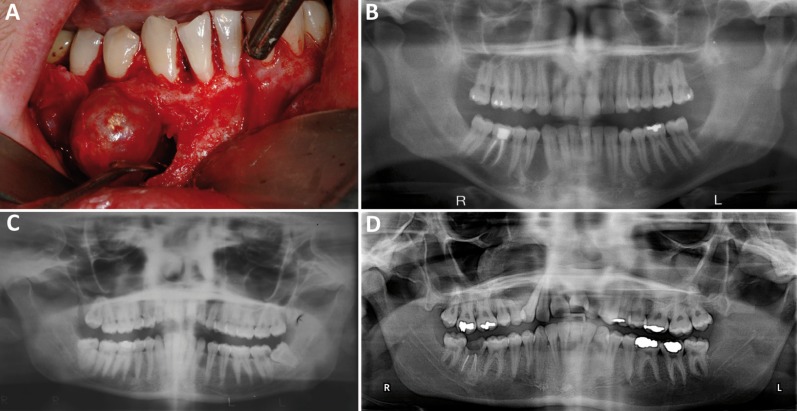
Radiological features of three Extra-Follicular AOT cases. A: This clinical photograph is taken at surgery. Note the round soft tissue nodule emerging from the cavity between the right mandibular canine and first premolar. B: This panoramic image demonstrates a corticated unilocular radiolucency of the nodule emerging from the cavity in Figure A. C: This panoramic radiograph demonstrates small and well-demarcated unilocular radiolucency in the left mandible between the second premolar and canine in the higher portion of the root. D: This panoramic radiograph demonstrates a large unilocular radiolucency pushing the left maxillary second premolar and canine apart.

-Clinical Findings: Extra-Follicular AOT (present between teeth; Figs. 2a-2d)

Only three (22%) of the 14 AOT cases were extra-follicular in type. The age range for the extra-follicular AOT type was 19-30 (mean = 26); two were in females. Of the three, two were in the mandible (cases 1, 4) and one in the maxilla (case 8). Figure 2A shows the tumor in case 1 at surgery in form of an encapsulated smooth surfaced nodule of soft tissue emerging out of a bony cavity. Figure 2B is case 1 depicted radiographically in form of a unilocular radiolucency between teeth #s 27 and 28. Figure 2C (case 4) shows a unilocular radiolucency high up between teeth #s 21 and 22 radiographically simulating a lateral periodontal cyst. Figure 2D represents the third extra-follicular AOT between teeth #s 5 and 6 clearly pushing teeth apart.

-Clinical Findings: Peripheral AOT (gingiva)

Two (14%) of the AOT cases were peripheral in type. These occurred in one male and one female, 12 and 13 years of age respectively. Both presented as gingival swelling on the buccal gingiva of the anterior maxilla. Case 9 was between teeth #s 8 and 9 and case 14 was between teeth #s 7 and 8. Case 9 presented with bleeding; case 14 was described as soft.

-Radiographic Findings

All 12 intra-osseous AOT cases (9 follicular and 3 extra-follicular) presented radiographically as well-defined to corticated unilocular radiolucencies (Figs. 1,2). Only case 5 (Fig. 1A) showed specks of radiopacity within the radiolucency. Five of the nine follicular AOT cases showed some form of tooth displacement (cases 3, 5, 6, 7 and 13, depicted in Fig. 1A-D). Case 13 (Fig. 1D) shows the radiolucency interfering with the eruption of tooth #22. Cases 1 and 8 (Fig. 2B,D) of the three extra-follicular AOT patients showed displacement of teeth. Case 8 (Fig. 2D) shows significant displacement of tooth #6, causing three-plus mobility of the tooth which had to be extracted for lack of bony support. The third case in this category, case 4 (Fig. 2C), showed very little to no tooth displacement with radiographic features simulating a lateral periodontal cyst. There were no radiographic changes associated with the two peripheral AOT cases 9 and 14.

-Pathologic Findings

**Figure 3:**
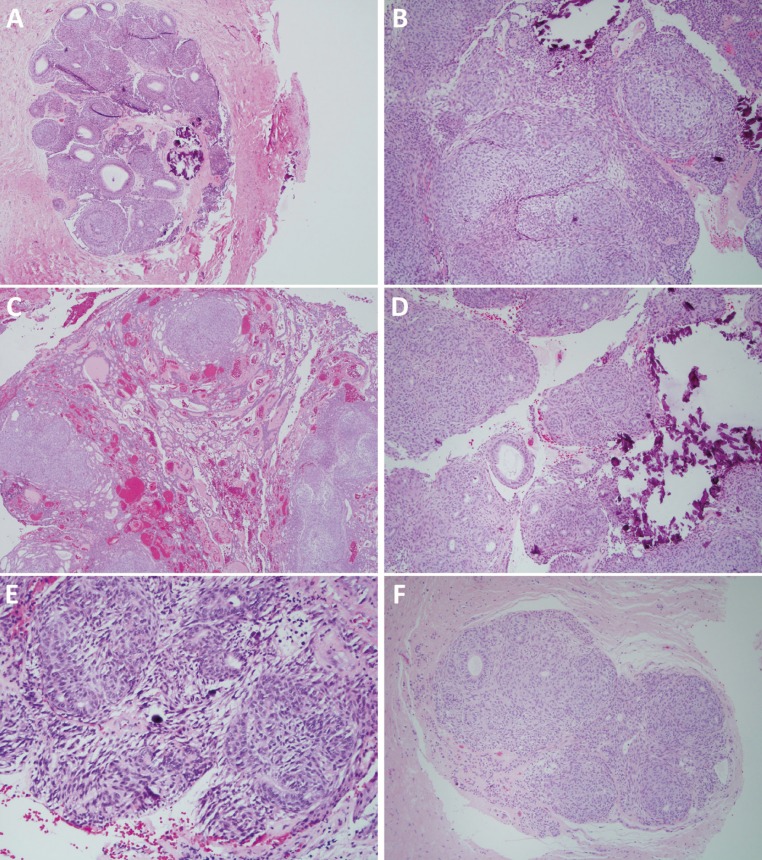
Histological features of all 14 AOT cases. A: H & E stained section at x 100 magnification demonstrating a thick capsule, many duct-like structures lined by one layer of cuboidal epithelial cells and some calcifications. B: H & E stained section at x 100 magnification demonstrating a solid tumor made up of whorls and spheres of epithelial cells suspended on scant connective tissue background. Note small clusters of calcifications. C: H & E stained section at x 100 magnification demonstrating thin cords of epithelial cells suspended on vascular connective tissue background. D: H & E stained section at x 100 magnification demonstrating large aggregates of calcifications along with solid epithelial whorls and a few duct-like structures. E: H & E stained section at x 100 magnification demonstrating the histology of one of the two peripheral AOT cases. Note a few duct-like structures, epithelial whorls and small cementum-like globules of calcified material. F: H & E stained section at x 100 magnification demonstrating the histology of the second peripheral AOT case. Note the tumor surrounded by the gingival connective tissue. The tumor is made up of solid epithelial whorls and a few duct-like structures.

Figure 3 (A-F) represents the histological features of all the 14 AOT cases. Figures 3A-D represent the main histologic features of the 12 intraosseous AOT cases (follicular and extra-follicular) while Figures 3E and 3F represent the histological features of the two peripheral AOT cases. The histology was defined by the presence of a thick capsule (Fig. 3A), duct-like structures lined by one layer of cuboidal cells (Fig. 3A), whorls or spheres made up of spindle-shaped epithelial cells (Fig. 3B), thin and elongated cords of epithelial cells (Fig. 3C), and calcified material including cementum-like globules (Fig. 3A-B, D-E). All of the 14 cases (100%) showed some degree of duct-like structures as depicted in Figures 3A and 3D-3F. Duct-like structures were rare in case 5 but numerous in cases 3, 7 and 11. All of the cases (100%) showed whorls or spheres of epithelial cells (Fig. 3A-F). Nine of the 14 cases (64%) showed elongated and slender cords (Fig. 3C), giving the tumor a loose and cystic morphology. Cases 3, 9, 11, 12, and 14 were mostly solid tumors. Eleven of the 14 cases (79%) had calcified material (Fig. 3A-B,D-E), including one peripheral AOT (case 14, depicted in Fig. 3E). Calcified material was absent in cases 2, 6 and 9. Twelve of the 14 cases (86%) showed evidence of a capsule (Figure 3A) ranging from thin to thick. The two peripheral AOT cases were surrounded by the connective tissue of the overlying gingiva and no evidence of a true capsule was identified (Fig. 3E,F). The epithelial component in all cases was suspended on a scant background of connective tissue. Case 2 (Fig. 3C) had prominent vasculature.

## Discussion

Adenomatoid odontogenic tumor is an uncommon benign neoplasm of odontogenic epithelial origin constituting less than 3% of all odontogenic tumors (1-3). At the OPBS of a major university, AOT cases accounted for 0.03% of the biopsies in a nine-year period, rendering it a rare condition worthy of reporting and examining. We present three types of AOT: follicular, constituting 64% of the cases, compared to a rate of 75% in the literature; extra-follicular, constituting 22% of the cases similar to that in the literature of 25% (1). In our study, the peripheral AOT cases constituted 14% of our cases which is much higher than the reported 5% (1). Previously, the literature characterized AOT as a tumor that occurs in the anterior maxilla at a ratio close to 2:1 and at a ratio of almost 2:1 in females in the second decade of age. In this seasoned literature, it is described as appearing in association with impacted canines 60% of the time (1,10,12). Others reported that two-thirds of these cases occur in the anterior maxilla, two-thirds occur in young females, and two-thirds present with impacted canines (3,9). Yet, the more recent literature shows a shift in the typical characteristics of this tumor, especially with regard to site; the anterior mandible has more recently been suggested as the most common site for this condition (3,9,12).

With regard to site, if we take all 14 cases, the ratio of clinical site prevalence in maxilla to that in the mandible is 1.3:1. If we exclude the two peripheral AOT cases, the ratio of occurrence in the maxilla to that in the mandible in the 12 intraosseous cases is 1:1. The nine follicular cases demonstrate a maxilla-to-mandible prevalence ratio of 1.3:1, similar to that of the whole. The extra-follicular cases demonstrate a maxilla to mandible ratio of 1:2. The two peripheral cases were exclusively in the anterior maxilla. This academic institution’s experience adds another dimension to the recent change suggested by Sethi *et al.* (17). We present new data to the literature proposing that occurrence in the anterior maxilla is slightly more common or at least at an equal rate to that in the anterior mandible in AOT cases that occur in bone.

With regard to clinical gender prevalence, when the 14 cases are measured as a whole, the female-to-male ratio is also 1.3:1. The female-to-male ratio remained constant with the nine follicular cases (1.3:1), but changed with the three extra-follicular cases (2:1). The gender ratio of the two peripheral cases was 1:1. Again, our study presents a lower gender ratio than those that report a nearly 2:1 female-to-male ratio (18).

The age range in our study was 11-30 with a mean age of 16, consistent with what is described in the literature, which also reports a mean age of 16 years (1,18). Our study shows that all the follicular and the peripheral AOT cases occurred in patients under 20 years of age. The age range for the follicular AOT cases was 11-17 (mean 13) and the two peripheral cases were in patients who were 12 and 13, confirming the notion that most AOT cases tend to occur in the second decade of life (1). The oldest patients in this study were 28 and 30 years of age and both had the extra-follicular AOT type, consistent with some reports suggesting that extra-follicular AOT cases tend to occur in patients older than those in the other two AOT types (3,7,12).

Association with impacted canines is also a point that is highlighted in the literature, which indicates that 60% of all AOT cases present in association with impacted canines (10). In our study, when the 14 cases are taken as a whole, 36% of cases are associated with impacted canines, a rate lower than that reported. However, when the 2 peripheral AOT cases are excluded and the 12 intraosseous AOT cases are measured separately, the rate of association with impacted canines goes up to 42%, still lower than that reported. The incidence goes up further when follicular AOT cases are measured separate from the other two AOT types; by this count, 56% of cases are associated with impacted canines, consistent with that reported. In the follicular AOT cases, three of five (60%) cases are associated with the maxillary canines.

There is a unique aspect to the clinical presentation of some of the cases in this study, where a spectrum of clinical presentation is highlighted. The clinical presentation ranges from typical nonaggressive mild swelling (10 cases) to aggressive behaviour (one case) and clinically unnoticed (one case). Case 8 acted aggressively, pushing teeth apart (Fig. 2D) and destroying bone to the point of leaving tooth #6 hanging in space with no bony support and three-plus mobility. The tooth could not be saved and had to be extracted at surgery. On the other hand, case 4 (Fig. 2C) was not noticed clinically and was discovered radiographically as an incidental finding. These findings are reported elsewhere (19) but are rare. The last clinical point worth highlighting is the color of the intra-osseous AOT; nearly all the follicular and extra-follicular cases were covered by pink mucosa. The exception was case 2 that was clinically described as purple in color. To our knowledge, this has not been described before and; its clinical significance is unknown. It is possible that this tumor was more vascular and the lesion was closer to the thin buccal bone than usual.

Radiographically, all 12 follicular and extra-follicular cases presented as unilocular radiolucencies with corticated borders. Only one case showed specks of radiopacity (Fig. 1A). It is important to state that histologically, 11 of 14 (79%) cases showed some degree of calcification, but apparently this was not enough to show radiographically. The radiographic presentation of the 12 cases is similar to what is described in the literature, confirming the slow and benign behaviour of this neoplasm. Tooth resorption was not noted in any of the 12 cases.

Histologically, all 14 cases were diagnosed based on morphologic features present on the H&E–stained slides as depicted in Figure 3 (A-F). All cases displayed histological features typical of AOT similar to that described in the literature. Eleven of the 14 cases (79%) had some calcification, similar to the reported rate of 78% (20). None of the 14 cases showed any evidence of amyloid.

All 14 cases were conservatively surgically treated with enucleation and curettage, the treatment of choice for AOT. In follicular AOT cases, there is a role for marsupialization to save and allow the impacted tooth to erupt with concomitant orthodontic assisted guided eruption (21). If clinically applicable, the tooth in the intraosseous follicular type of AOT should be saved and orthodontically helped to erupt. For the other two AOT subtypes, the extra-follicular and peripheral, most surgical plans should be noninvasive and conservative (22).

In conclusion, we present 14 AOT cases, an uncommon odontogenic tumor, representing 0.03% of 45,667 cases diagnosed over nine years in a large academic OPBS. We present these cases in great clinical detail in an effort to share diagnostic features that can assist pathologists and clinicians who will, at some point, need to diagnose and treat patients with AOT, without the benefit of having personally encountered other such cases previously.
